# Acceptability Study on HIV Self-Testing among Transgender Women, Men who Have Sex with Men, and Female Entertainment Workers in Cambodia: A Qualitative Analysis

**DOI:** 10.1371/journal.pone.0166129

**Published:** 2016-11-09

**Authors:** Khuondyla Pal, Chanrith Ngin, Sovannary Tuot, Pheak Chhoun, Cheaty Ly, Srean Chhim, Minh-Anh Luong, Brent Tatomir, Siyan Yi

**Affiliations:** 1 KHANA Center for Population Health Research, Phnom Penh, Cambodia; 2 Center for Global Health Research, Public Health Program, Touro University California, Vallejo, California, United States of America; 3 Population Services Khmer (PSK), Phnom Penh, Cambodia; 4 FHI 360, Phnom Penh, Cambodia; Katholieke Universiteit Leuven Rega Institute for Medical Research, BELGIUM

## Abstract

**Background:**

In Cambodia, HIV prevalence is high while HIV testing rates remain low among transgender women (TG women), men who have sex with men (MSM), and female entertainment workers (FEW). Introducing self-testing for HIV to these key populations (KPs) could potentially overcome the under-diagnosis of HIV and significantly increase testing rates and receipt of the results, and thus could decrease transmission. Therefore, this study aimed to determine the acceptability of HIV self-testing (HIVST) among these three categories of KPs.

**Methods:**

This study was conducted through focus group discussions (FGDs) with TG women, MSM, and FEW in Phnom Penh city, Kampong Cham, Battambang, and Siem Reap provinces of Cambodia. Convenience sampling was used to recruit the participants. Two FGDs (six participants in each FGD) were conducted in each target group in each study site, totaling 24 FGDs (144 participants). Thematic analysis was performed to identify common or divergent patterns across the target groups.

**Results:**

Almost all participants among the three groups (TG women, MSM, and FEW) had not heard about HIVST, but all of them expressed willingness to try it. They perceived HIVST as confidential, convenient, time-saving, and high-tech. Barriers to obtaining HIVST included cost, access, administration technique, embarrassment, and fear of pain. The majority preferred counseling before and after testing.

**Conclusions:**

Participants showed high willingness to use and acceptability of HIVST due to its confidentiality/privacy and convenience even if it is not linked to a confirmatory test or care and treatment. Notwithstanding, to increase HIVST, the target groups would need affordable self-test kits, education about how to perform HIVST and read results, assurance about accuracy and reliability of HIVST, and provision of post-test counseling and facilitation of linkage to care and treatment.

## Introduction

HIV self-testing (HIVST), a process in which an individual performs an HIV rapid diagnostic test (RDT) and interprets the result in private [1, 2], was first proposed in the mid-1980s [3]. HIVST is an emerging approach that is well accepted, potentially cost-effective, and empowering for those who may not otherwise test, particularly key populations (KPs) [2, 4–6].

Globally, KPs, including men who have sex with men (MSM), sex workers, people who inject drugs (PWID), and transgender people, collectively have the HIV prevalence 10–50 times greater than the general population [7, 8]. Approximately 40% of all new HIV infections each year are among the KPs [7, 8]. Cambodia is no exception to this trend. The National Centre for HIV/AIDS, Dermatology and STD (NCHADS) estimated that the overall HIV prevalence rate among the general adult population in Cambodia was 0.28% in 2015. However, the HIV prevalence rate was higher among KPs. High HIV prevalence rates were recorded among transgender women (TG women) (5.9% in 2016), MSM (2.3% in 2014), and female entertainment workers (FEW) who had more than seven sexual partners per week (14% in 2010) and those who had seven or less sexual partners per week (4.1% in 2010) [9–11].

Individuals who are unaware of their HIV status have a transmission rate of 3.5 times higher than individuals who are aware of their status [1]. Although research shows that detecting HIV infection is a key to reducing HIV transmission [12–14], HIV testing rates remain low among KPs in Cambodia. According to KHANA’s program report, 51,511 members of KPs were reached between January 2014 and May 2015 and 52.2% of them had previously been tested through finger-prick by KHANA’s implementing partners during this period. Moreover, to achieve UNAIDS’ “90-90-90” targets (90% of people with HIV knowing their status, 90% linked to anti-retroviral therapy “ART”, and 90% virally suppressed) [15], more concerted efforts are needed to improve the uptake of HIV testing among KPs. HIVST is believed to remove several concerns related to privacy and confidentiality, and increase the potential to reach individuals who have limited access to testing services [1].

Many countries have considered implementing HIVST to overcome the under-diagnosis of HIV [8, 16–20] because it significantly increases testing rates and receipt of the results [21, 22]. Cambodia is considering implementing this new approach to increase the test uptake and achieve the UNAIDS’ “90-90-90” targets. No previous study has been conducted on the acceptability of HIVST in Cambodia. Therefore, this study aimed to determine the acceptability of HIVST among FEW, MSM, and TG women in Cambodia. Findings from this study will be used to explore the possibility of implementing HIVST and to prepare for potential challenges.

The purpose of this study was to assess the awareness, acceptability, and perceptions of HIVST among TG women, MSM, and FEW in Cambodia. The objectives of this study were:

▪ To assess the awareness of HIVST among TG women, MSM, and FEW.
▪ To understand the acceptability and perceptions of HIVST among TG women, MSM, and FEW.
▪ To identify the need of counseling and the counseling processes which can link HIVST to HIV services for TG women, MSM, and FEW who test positive.

## Materials and Methods

### Ethical statement

Participation in this study was voluntary, and a written informed consent was obtained from each study participant after a detailed description of the study objectives and procedures was explained to the participants. Moreover, the study participants had an opportunity to refuse or discontinue participation at any time. Privacy was strictly protected by conducting the FGDs at a private place, and we ensured confidentiality of the respondents by removing all personal identifiers from the FGD transcripts. The study protocol was approved by the National Ethics Committee for Health Research (NECHR) of the Ministry of Health, Cambodia (No. 282 NECHR).

### Study sites and sampling

This study was conducted in September 2015 in the towns of Phnom Penh city, Kampong Cham, Battambang, and Siem Reap provinces, which have the largest numbers of TG women, MSM, and FEW in Cambodia. The investigation was qualitative in design, and was conducted through focus group discussions (FGDs) with these KP members.

Each FGD included six participants. In each study site, two FGDs were conducted with each target group, totaling 24 FGDs (144 participants). Convenience sampling was used to recruit the participants.

All participants were at least 18 years old, spoke Khmer, and were able and willing to provide a written informed consent. TG women had to be biologically male, self-identify as a female, and self-report having sex with at least one man in the past 12 months. MSM had to be biologically male, self-identify as MSM, and self-report having sex with at least one man in the past 12 months. FEW participants had to self-identify as a FEW, which meant being currently employed at an entertainment establishment, and self-report having sex with a least one man in the past 12 months.

### Data collection training and procedures

Data were gathered by a team who had gone through one-day training on the study protocol, research ethics, how to conduct FGDs, and data collection procedures. The training was provided by the key researchers.

Participants associated with NGOs were recruited by the data collection team, with assistance from outreach workers or field staff from NGO partners and respondent networks. The outreach workers/field staff and data collection team visited locales associated with TG women, MSM, and FEW to discuss the study with potential participants and recruit them to take part in the FGDs.

All FGDs were conducted in private locations. The data collectors acted as moderators and note takers. Videos of blood and oral HIVST were shown to the participants before they were asked to express their opinions about the two types of HIVST. The researchers observed the discussions, and audio recorders were used to tape all FGDs in order to ensure the accuracy of the participants’ views. Each FGD lasted approximately 60–90 minutes.

### Instrument development

Discussion guides were developed by the research team, which included instructions and prompt questions to ensure the flow and consistency during the FGDs. The questions were modified from a previous published paper [14], and were pre-tested among the three populations for content validity and understandability of the language used. The questions asked about the participants’ socio-demographic characteristics; awareness, acceptability, and perceptions of HIV self-test; and its integration with linkages to care which engages newly diagnosed HIV-infected persons into HIV care and treatment.

### Data analyses

The research team transcribed all recorded FGDs. The transcripts were coded using Nvivo version 7. The electronic codes were transferred to a Word file. Then, a common narrative was developed by using recurring themes identified during data analyses. Thematic analysis was performed to identify common or divergent patterns across the target groups.

## Results

### Characteristics of participants

In total, 144 respondents participated in the FGDs. The mean ages of TG women, MSM, and FEW were 25.9, 26.9, and 27.7 years, respectively. Of the 48 TG women, 64.6% considered themselves female and 35.4% considered themselves third gender. The majority (91.7%) of TG women was never married, 4.2% were married, and 4.2% were widowed/divorced/separated. With regards to schooling, 4.2% of TG women had no education, 20.8% had a primary education, 66.7% had a high school education, and 8.3% had a higher education. Concerning their work, 70.8% of TG women had their own business, and 14.6% worked in a private company. The reported average monthly income of this group was $179.3.

Of the 48 MSM participants, 52.1% considered themselves male and 47.9% considered themselves third gender. All of them were never married. As for schooling, 6.2% of MSM had no education, 16.7% had a primary education, 62.5% had a high school education, and 14.6% had a higher education. Regarding their work, 33.3% of MSM had their own business, and 29.2% worked in a private company. The reported average income of this group was $119.3.

Of the 48 FEW participants, 20.8% were never married, 27.1% were married, and 52.1% were widowed/divorced/separated. On schooling, 10.5% of FEW had no education, 43.7% had a primary education, and 45.8% had a high school education. About 46% of FEW worked in karaoke parlors, and 29.2% worked in restaurants. The reported average income of this group was $279.8.

### Awareness about HIV self-test among TG women, MSM, and FEW

Virtually, all participants had never known about HIVST. Only two out of the 48 TG women had heard of HIVST from friends, and none of the MSM and FEW participants had heard of HIVST. All participants understood the basic meanings of HIV positive and HIV negative results.

### Acceptability of HIV self-test, need of counseling, and perceived barriers to linkage to care among TG women

All TG women wanted to try HIVST. Some preferred trying both oral and blood tests. They described HIVST as confidential, easy, fast, self-administered, time-saving, and high-tech.

“It (HIVST) is easy, and does not require seeing a doctor. Moreover, it is confidential. If the result is positive, we go to consult a doctor and get ART.” -A TG woman in FGD in Siem Reap province

Barriers to obtaining HIVST for the TG community included a lack of knowledge surrounding cost, access, administration technique, as well as shyness, fear, pain, and embarrassment. In the past, the majority of TG women in this study had experienced voluntary confidential counseling and testing (VCCT) and finger-prick testing either at health facilities or through NGOs’ outreach workers, and said they would prefer HIVST to VCCT. All TG women thought HIVST was a reliable and confidential method. They would use HIVST if it were free, but also believed that a range of $1.25-$10 (mean = $3.1 and median = $2.5) would constitute an acceptable price. They suggested that HIVST kits should be sold at pharmacies, hospitals, clinics, health centers, NGOs, NGO clubs, and marts (or shops).

The majority of TG women said they would need counseling before and after testing, including how to use HIVST. Moreover, the pre- and post-test counseling was not necessarily done in person, but could be through hotline phone calls. They understood that there were multiple avenues for counseling. When asked what to do if the result was positive, TG women said they would go to a health center, begin ART, and seek advice, counseling, confidentiality, and support.

“…I would follow the doctor’s advice after post-counseling (about the positive result). I would seek ART and make a life plan to live like others. I would not commit a suicide. After the post-counseling, I would face the reality.”–A TG woman in FGD in Phnom Penh city

Also, they said no job termination should occur, the result should be confirmed and kept confidential, and the patient should take action to prevent further transmission. However, barriers to treatment, if infected, included fear, shyness, embarrassment, discrimination, denial, loss of self-efficacy, and a lack of knowledge surrounding how and where to receive treatment. If the result was negative, TG women said they would work to prevent future infection with more protection.

### Acceptability of HIV self-test, need of counseling, and perceived barriers to linkage to care among MSM

All MSM wanted to try HIVST. They described HIVST as confidential, easy, fast, self-administered, time-saving, requiring less people, reliable, and convenient.

“…It (HIVST) is time-saving, and does not require going to a public facility, such as a hospital or clinic. Moreover, only we know about it (the result)…At a testing facility we could see people (we know), so it is bothersome.”–A MSM in FGD in Kampong Cham province

Barriers to obtaining HIVST for the MSM community included a lack of knowledge surrounding cost, access, administration technique, reliability, as well as shyness, discrimination, and absence of Khmer translation for the instructions. In the past, the majority of MSM had experienced VCCT and finger-prick testing either at health facilities or through NGOs’ outreach workers, and said they would prefer HIVST to VCCT, with half thinking it was reliable. If the result was positive, they would seek confirmation with an additional administration. They would use HIVST if it were free, but if there was a price attached, they said they would prefer to go to a hospital for a free test. They believed that a range of $0.5-$10 (mean = $3.7 and median = $1.5) would constitute an acceptable price. They suggested that HIVST kits should be sold at pharmacies, clinics, hospitals, health centers, markets (they approved of advertisement), and places where condoms are sold.

The majority of MSM said they would need counseling before and after testing, including how to use HIVST. They understood that there were multiple avenues for counseling. When asked what to do if the result was positive, MSM said they would go to a health center, begin ART, and seek advice, counseling, confidentiality, and support.

“…If the result is positive, we should go to a place where we can consult because normally we just know the result, but we do not know how to take care of our health. Only an NGO or a health center can advise us about it.”–A MSM in FGD in Kampong Cham province

If infected, they would reduce the number of partners, use condoms, plan for their future, and take action to prevent further transmission. Barriers to treatment, if infected, included fear, shyness, embarrassment, discrimination, cost, access to a healthcare facility or physician, and bad previous interactions with physicians. If the result was negative, they would work to prevent infection with more protection and regular check-ups.

### Acceptability of HIV self-test, need of counseling, and perceived barriers to linkage to care among FEW

All FEW wanted to try HIVST, but perceived blood HIVST to be more accurate and familiar than oral HIVST. However, they thought that oral HIVST would be easy and good for patients scared of needles. They described HIVST as confidential, easy, fast, self-administered, time-saving, not requiring a physician, reliable, safe (less chance of HIV transmission), and discreet (good for use if an individual doesn’t trust a partner).

“It (HIVST) is time-saving and not tiring. Also, it saves transport cost, such as Motordup (taxi motorbike) fare (for going to a facility). Facility-based testing is time-consuming and requires lots of counseling. HIVST is fast.”—A FEW in FGD in Battambang province

Barriers to obtaining HIVST for the FEW included a lack of knowledge surrounding cost, access, administration technique, reliability, as well as shyness, HIV transmission risk, and fear. In the past, the majority of FEW had experienced VCCT and finger-prick testing either at health facilities or through NGOs’ outreach workers, and said they would prefer HIVST to VCCT, contending that it was reliable. Some of them would use HIVST if it were free; but others would not use a free kit because they believed it would be a poor-quality product. Despite a few outliers, the majority believed that a range of $0.5-$12 (mean = $4.2 and median = $3) would constitute an acceptable price. Some mentioned that price did not matter because safety was more important. They suggested that HIVST kits should be sold at pharmacies, hospitals, clinics, health centers, NGOs, NGO clubs, and places where they were working.

All FEW said they would need counseling before and after testing, including how to use HIVST. They understood that there were multiple avenues for counseling. When asked what to do if the result was positive, FEW said they would go to a health center, begin ART, get check-ups, and seek counseling, confidentiality, and support. Barriers to treatment, if infected, included fear, shyness, embarrassment, discrimination, cost, denial, and wanting status to remain private. If the result was negative, they would seek advice for infection prevention.

## Discussion

HIVST represents great potential to optimize HIV testing and possibly connection to care and treatment among hard-to-reach key populations. Extensive reviews of HIVST studies in developing countries indicate that individuals who self-test are likely to use HIVST for their next HIV test and indicate they would recommend HIVST to family and friends [2,7]. Notwithstanding, HIVST should be complementary to facility- and community-based HIV testing and counseling services.

This study depicts high acceptability of HIVST among the key populations in Cambodia, which is consistent with findings in other studies [2,7]. Almost all participants from the three groups preferred self-test mainly because it provides confidentiality (or privacy), saves time, and is convenient, while they articulated several concerns regarding the cost, administration of the test, reliability and accuracy of the test, and handling of HIV positive result.

These findings are analogous to those of other studies that stipulate that convenience and privacy are the prime drivers for acceptability of HIVST among key populations [2, 7]. Convenience and privacy would also incentivize them to have better control over their health [23] and increase testing frequency [24].

The concern about the cost implies that price is an important factor in determining the uptake of self-test, which is a common constraint in poor-resource settings [2, 7]. While the participants were willing to use a self-test, a certain number across the three groups said they would not use it if the price was high (i.e., > $0.5), and would use a free service from NGOs instead. In Cambodia, HIV testing services have been widely offered free of charge at VCCT centers run by public facilities and NGOs, and in communities through an extensive network of community support volunteers and peer outreach workers who work closely with key populations. Therefore, some of these groups may be unwilling to pay even a nominal price for HIVST kits.

Media advertising may also serve as an important resource to increase awareness of HIV self-test by these key populations. Those willing to pay reported the self-test kits should be sold at pharmacies, hospitals/clinics, NGOs and NGO clubs, marts (or shops), places where condoms are sold, and their work places (i.e., entertainment establishments for FEW).

Concern about the accuracy of self-test was also expressed, paralleling findings in other studies [2,7]. Therefore, research on accuracy and reliability of test results performed by skilled personnel and lay persons is necessary to avoid false positive or negative results, and to provide confidence among self-testers. Further, once the data suggesting reliability exists, dissemination of these results to the key populations will be important.

Fear of pain and how to properly administer the test were other concerns among the target groups. These concerns may stem from the novel nature of HIVST technology in these communities and their limited functional literacy. Test administration requires a certain level of literacy to ensure confidence in interpreting results. Providing a televised or social media tutorial on a correct self-test procedure may be beneficial for the target populations, promote visibility of a self-testing strategy, and increase users’ confidence.

The majority of participants thought it is essential to have pre- and post-test counseling. This is in line with other findings in low-income countries [2,7]. The study subjects suggested counseling be provided in different ways, such as face-to-face or over a phone before purchasing, or through hotline/telephone calls-in before and after purchasing. Although the vast majority of the participants agreed that self-test should be publicly sold, the challenges of implementing self-test would include providing a confirmatory test and referral for individuals with positive results. Seeking post-test counseling and referral to treatment normally is the end users’ responsibility. Provision of post-test counselling and facilitation of linkage to care and treatment would ergo expand self-testing among these key populations. Health centers were mentioned as a preferred facility to go to if a positive result was determined. Refining back-up testing and counselling services at health centers would consequently encourage the target groups to self-test.

This study was the first to examine the awareness, acceptability, and concerns of HIVST among key populations in Cambodia; and it holds a few limitations. First, actual testing was not done. The participants provided overall ideas on the nature and use of HIVST. Better perceptions about HIVST may have been captured if the participants had performed a self-test as part of the study. Second, because this study was qualitative in design, it may benefit from a quantitative study to fill in the gaps in numbers and percentages. Third, future research should delve into how key stakeholders, such as health care workers, program implementers, and policy makers, view HIVST in light of testing uptake by key populations. Finally, real users’ linkage to care and treatment following a positive result is also worth investigating.

## Conclusions

In the Cambodian setting, HIVST could be an innovative mode to increase testing given the limited acceptance of testing among key populations. This study shows that the target groups (TG women, MSM, and FEW) expressed high willingness to use and acceptability of HIVST due to its confidentiality/privacy and convenience even if it is not linked to a confirmatory test or care and treatment. To increase HIVST, nonetheless, a number of their concerns should be tackled. First, self-test kits should be made available to these key populations at a price that is reasonable and affordable for them. Second, dissemination about how to do HIVST and read results, such as via TV and social media, should be conducted to bolster confidence among self-testers. Research on accuracy and reliability of HIVST would also enhance attraction and confidence among these potential clients. Finally, provision of post-test counseling and facilitation of linkages to care and treatment, particularly at health centers, should be done to attract and retain users, and ultimately to maximize the merit of HIVST.
